# Entropy-based detection of Twitter echo chambers

**DOI:** 10.1093/pnasnexus/pgae177

**Published:** 2024-04-25

**Authors:** Manuel Pratelli, Fabio Saracco, Marinella Petrocchi

**Affiliations:** IMT School For Advanced Studies Lucca, Piazza San Francesco 19, Lucca 55100, Italy; Istituto di Informatica e Telematica, CNR, via G. Moruzzi 1, Pisa 56124, Italy; “Enrico Fermi” Research Center, Via Panisperna 89A, Rome 00184, Italy; IMT School For Advanced Studies Lucca, Piazza San Francesco 19, Lucca 55100, Italy; Institute for Applied Computing “Mauro Picone”, CNR, Via dei Taurini 19, Rome 00185, Italy; Istituto di Informatica e Telematica, CNR, via G. Moruzzi 1, Pisa 56124, Italy; IMT School For Advanced Studies Lucca, Piazza San Francesco 19, Lucca 55100, Italy

**Keywords:** echo chambers, misinformation, Twitter, complex networks, information theory

## Abstract

Echo chambers, i.e. clusters of users exposed to news and opinions in line with their previous beliefs, were observed in many online debates on social platforms. We propose a completely unbiased entropy-based method for detecting echo chambers. The method is completely agnostic to the nature of the data. In the Italian Twitter debate about the Covid-19 vaccination, we find a limited presence of users in echo chambers (about 0.35% of all users). Nevertheless, their impact on the formation of a common discourse is strong, as users in echo chambers are responsible for nearly a third of the retweets in the original dataset. Moreover, in the case study observed, echo chambers appear to be a receptacle for disinformative content.

**Significance Statement**

While the concept of echo chambers is clear, a generally accepted method for their detection is still lacking in the literature. Our study provides a general and unbiased method for detecting echo chambers, using entropy-based null models as statistical benchmarks. The rationale is to detect groups of users with similar opinions based on the significant similarity of the main content creators and, similarly, to detect groups of users engaged with the same news. A nontrivial overlap between the two groups indicates the presence of an echo chamber. Using the Italian Twitter debate on the Covid-19 vaccination as a case study, we found that users in echo chambers, while representing a small minority, strongly contribute to the debate, often disseminating misinformation.

## Introduction

In the virtual world, the tendency to seek out information that confirms existing beliefs and to interact with users who share similar opinions leads to the formation of echo chambers, i.e. “bounded, enclosed media spaces that have the potential to both amplify the messages delivered within it and insulate them from rebuttal” (1–4). We thus have two key events in echo chamber formation: (i) interaction between users with similar opinions; (ii) exposure of users to the same news articles.

##  

### Contributions

This article studies the actual presence of echo chambers in social networks by detecting the overlap of the two events. The detection is done by adopting an entropy-based technique. The platform considered in this study is Twitter/X. From now on, we will refer to the platform by its former name, Twitter, since the analyses were conducted before the change in the company name to X.

**Fig. 1.**
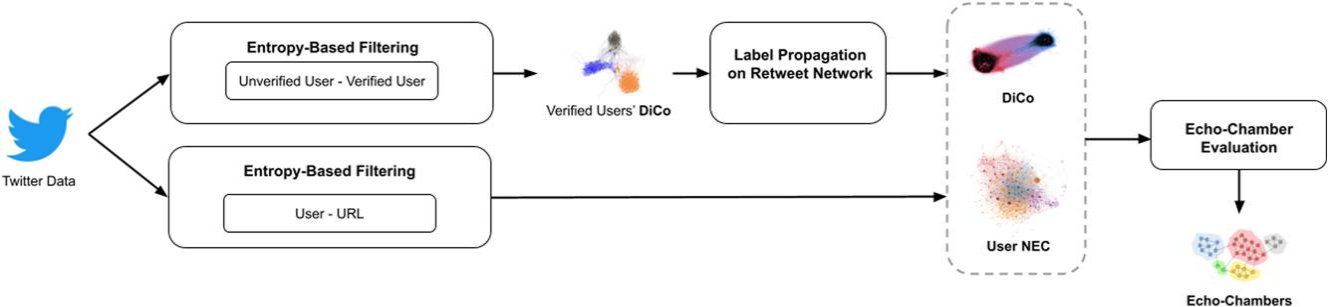
Pipeline for Echo-chamber detection. The upper path focuses on the detection of Discursive Communities (*DiCo*), while the lower one on the detection of News Engagement Communities (*NEC*). Both procedures pass through the statistical validation of empirical data with an entropy-based null model.

Recently, entropy-based null models have been introduced in studies of complex networks as an unbiased benchmark capable of revealing nontrivial structures of real systems (5), and thus they represent the appropriate framework for our analysis. Figure 1 shows how we intend to assess the occurrence of the two events, and, consequently, the occurrence of the echo chamber.

Assessing the opinions of the various accounts is not an easy task, but it can be inferred from the interaction among the various accounts. In Refs. (6–9), a method to infer the presence of a discursive community, i.e. a group of accounts contributing to the formation of a common discourse, was presented. It is based on verified users, i.e. those accounts for which Twitter has a procedure to check the identity of their owners. Verified accounts mainly belong to politicians, journalists, and celebrities; usually, they are strong creators of contents (6, 7, 9). Verified users are among the greatest contributors to the formation of a common discourse. It is possible, then, to let similarities emerge among the content created by verified users, based on the behavior of their common audience in terms of retweets, since retweets are considered a measure of engagement (10–12). In detail, for each pair of verified users, the number of common retweeters is counted. If the number is statistically significant with respect to an entropy-based benchmark, it is validated and we project a link between the verified users’ pair. On the monopartite network of verified users thus obtained, we run a community detection algorithm to extract groups of similar verified users (i.e. the *Verified users’ DiCo*s in Fig. 1). Then, the various communities of verified users are labeled in terms of the users who belong to them (since the users are verified, it is possible, for example, to derive their political leanings and test a posteriori the resulting communities). At this point, the labels are extended to unverified users using a label propagation algorithm (13) on the entire retweet network—thus encompassing both verified and unverified users.

Once again, the use of the retweet network for label propagation is motivated by the fact that there is evidence that users belonging to communities in a retweet network share similar views (10–12). In the following, such communities will be called *discursive communities* (or DiCo), and their detection is sketched in the top path of Fig. 1. Discursive communities *embrace those users who contribute to the formation of a common discourse*.

Regarding the exposure to the same news articles, we approach its assessment by analyzing the ties between the users and the URLs present in their tweets and retweets. The bottom path of Fig. 1 shows the approach. The idea of leveraging the bipartite network of users and URLs was already considered in Ref. (14) for Facebook: in the present case, we translate the idea therein to Twitter. Again, the procedure goes through a comparison between observations and an entropy-based benchmark: if two users tweeted (or retweeted) the same URLs significantly more than the benchmark, we conclude that the two users share the same information diet in a statistically significant way. We can thus identify groups of users sharing the same URLs. In the following, user communities that passed the validation are called *news engagement communities* of users, for short user NEC s. User NECs contextualize the second event: exposure of users to the same news articles.

Now, we were able to identify groups of users exposed to the same news articles (user NEC ) and groups of users who share a common discourse (DiCo). Users who share a group of the first type and a group of the second type form an echo chamber, provided they interact with each other. The interaction for us is that of retweets since retweets are considered as a form of endorsement to the content created by others (6, 10–12). Verifying user interactions is an important step because accounts belonging to the same user NEC may either not belong to the same DiCo or, even in the case where they are in the same discursive community, may not interact with each other. In this sense, only users who (i) belong to the same user NEC and (ii) belong to the same DiCo and (iii) are connected, even indirectly, through retweets (i.e. they form a weakly connected component in the retweet network) can be said to represent an echo chamber.

As a case study for evaluating the presence of echo chambers, we consider the online debate on Twitter regarding the Covid-19 vaccination campaign. Surprisingly, compared to numerous examples found in the literature, we find a limited presence of echo chambers in the analyzed dataset, mainly due to the small dimensions of users’ NECs. Although the detected echo chambers are composed of a small number of users with respect to the total number of active users, they play a significant role in terms of retweet interactions, i.e. the echo chambers that emerged in the case study of the Covid-19 vaccination debate have a significant impact on the creation of a common and cohesive discourse that is not devoid of disinformation. Furthermore, users who belong to such echo chambers show the same ideas and opinions after years.

Summarizing, the main contribution of this article is a novel unbiased method for echo chamber detection. The procedure is based on the very definition of echo chambers and involves the application of an entropy-based null model to discard signals assimilated to noise.

### Research questions

Keeping in mind that our ultimate goal is to observe if and when discursive communities and NECs of users overlap, thus forming echo chambers, we organize the structure of the article to answer the following research questions (RQs):

- RQ.1: What are the characteristics of the discursive communities (DiCos) and of the news engagement communities of users (users’ NECs)? Are there users in common?;
- RQ.2: What is the relation between the emergent echo chambers and the presence of disinformation, if any?

## Results

### Dataset

Our dataset consists of ∼1.87 M public posts in Italian and 136 k users; nearly ∼220 k tweets contain URLs. We relied on the Twitter’s streaming API and data were collected from 2021 September 1st to September 24th. The data collection was keyword-based and related to the COVID-19 vaccination online debate. The keywords are compatible with chronicles regarding the vaccination debate in Italy at time of data collection. We remind the reader that the Twitter’s streaming API returns any tweet containing those terms in the text of the tweet, as well as in its metadata. It is worth noting that it is not always necessary to have each permutation of a specific keyword in the tracking list. For example, the keyword “COVID” would return tweets that contain also both “COVID19” and “COVID-19”. The keywords for the data collection are in Section S1.

Data were downloaded using the Twitter API access for researchers (available at the time of the download) and were analyzed following X/Twitter’s policy (available at the following url: https://twitter.com/en/privacy).

### Discursive communities

**Fig. 2.**
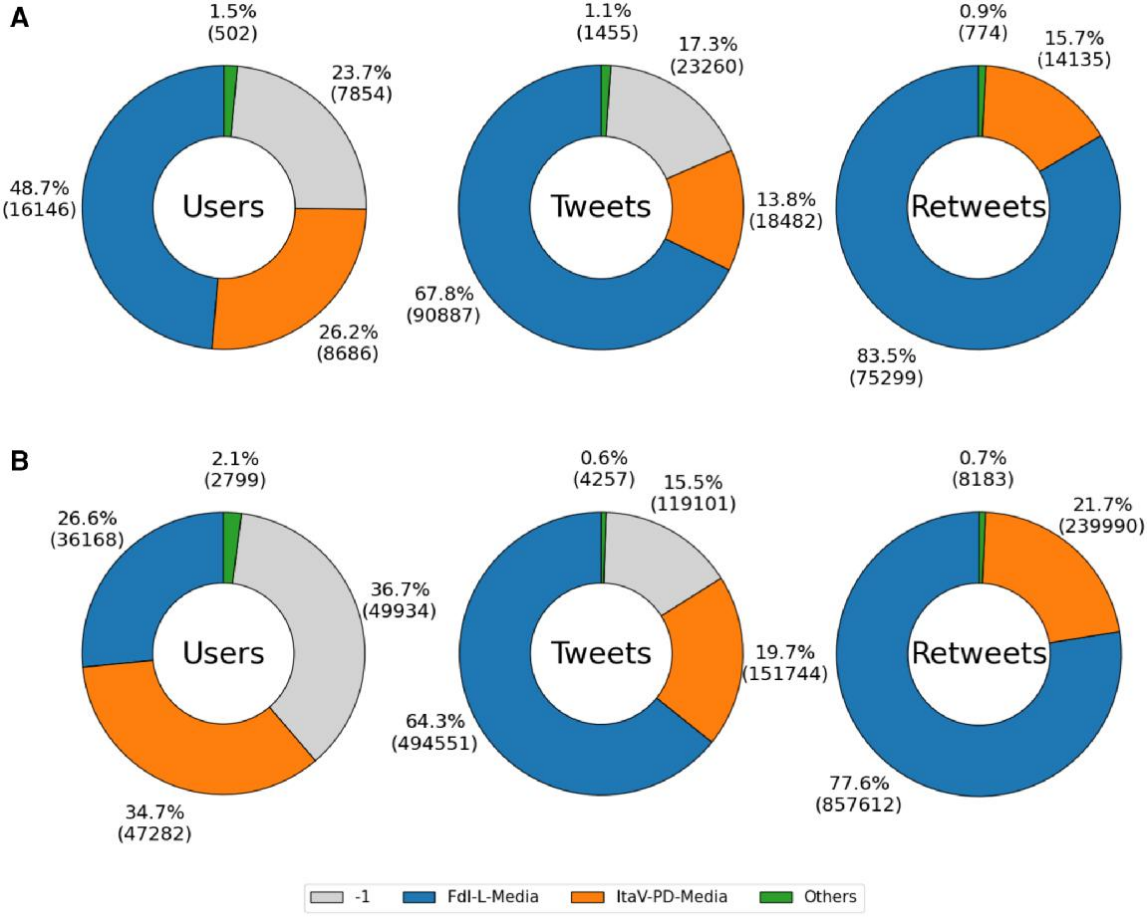
Characterization of the main DiCos in terms of the number of users, tweets, and retweets. The charts in A) consider all tweets and retweets, the charts in B) only consider tweets and retweets that contain URLs.

Figure 2(A) describes the characteristics of the main discursive communities (DiCos) that emerge from the data. We recall that it is possible to assign labels to verified accounts, as the identity of their owner has been certified by the platform. Starting from the original dataset, we run the community detection algorithm (15) on the validated network of verified users and the label propagation algorithm (13) on the network of retweets of the different communities. In our case study, two main discursive communities emerge, associated with political parties and Italian newspapers. Specifically, most of the users who are part of a DiCo belong either to the Itav-pd-media community (∼34.7%; the community includes journalists and exponents of the Italian parties Italia Viva and Democratic Party) or to the Fdi-l-media community (∼26.6%; the community includes journalists and exponents of the Italian parties Fratelli D’Italia and Lega). About 2.1% of users belong to smaller DiCos, while ∼36.7% of users do not belong to any DiCo. The Fdi-l-media community posted the most new content (64.3%), although it represents about a quarter of all users in our dataset. The Itav-pd-media community is responsible for 19.7% of the new content, while the remaining 15.5% is posted by users who do not belong to any particular community. In terms of retweets, Fdi-l-media is by far the most active community with 77.6% of the retweets.

Figure 2(B) characterizes DiCos by focusing only on posts containing URLs. In general, the observations made for the doughnut charts in panel (A) still hold, with the exception that almost half of the users who post tweets with URLs belong to the Fdi-l-media community (48.7%).

### NECs of users

Table 1 shows that of all users who have published at least one post with a URL (∼33k), only 566 are part of a user NEC, which is less than 2%. Accounts in user NECs are proportionally much more active in publishing URLs than users not validated by our procedure (67.7 vs 5.90 URLs per account).

**Table 1.** Users in user NEC.

Type	No. of users	Verified	No. URL
Nonvalidated	32,622	434	1,92,334
Validated	566	1	38,345

Validated users represent a limited minority of all accounts in the debate, i.e. less than 2% of all users that shared at least a URL.

**Fig. 3.**
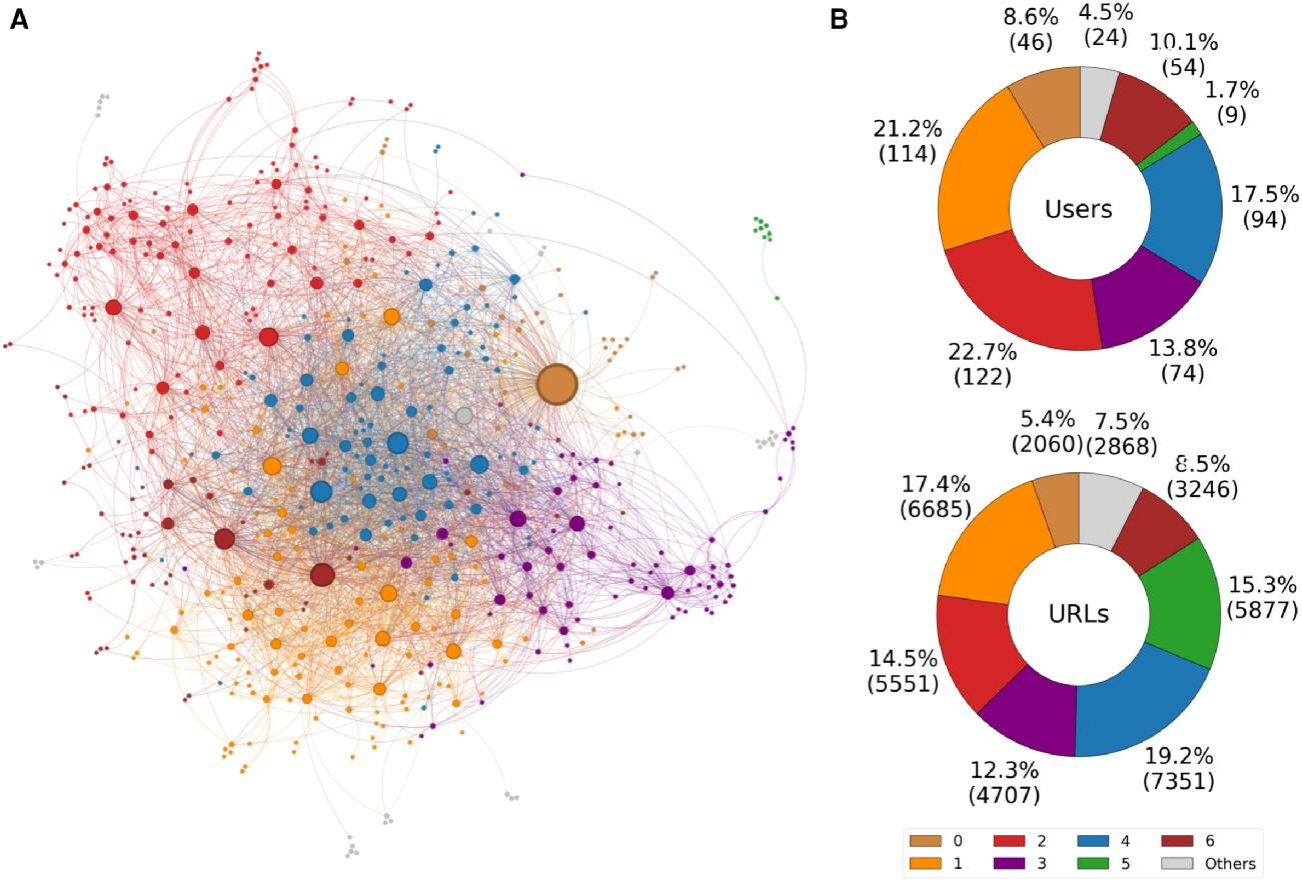
User NECs. A) Network representation of user NECs. B) Top: percentage (and number) of user NEC users belonging to each group. B) Percentage (and number) of URLs disseminated by users belonging to the various user NECs.

Figure 3(A) shows how the 566 users cluster into different user NECs, while Figure 3(B) provides a statistical view of the 566 users associated with the user NEC. On the right, the top doughnut chart illustrates the largest communities based on the number of users. Each of these prominent user NECs (IDs 0, 1, 2, 3, 4, 5, and 6) accounts for at least 95% of the total user population within this type of community. Furthermore, the lower doughnut chart shows that these communities have the highest frequency of tweets containing URLs. Communities 1, 2, 3, 4, and 5 collectively account for over 78% of the total URL traffic generated by all user NEC communities. An analogous analysis of the URL NECs, i.e. the community detected on the validated projection on the layer of the URLs can be found in Section S2.

### Echo chambers

Our analysis shows that all but 1 of the 566 users in the user NECs are also part of the same discursive community, i.e. Fdi-l-media. This is the discursive community with users affiliated with political parties Fratelli D’Italia and Lega, and news outlets showing similar leanings. However, the fact that all users in the user NECs belong to the same DiCo only tells us that users with similar “information diets” contribute to the formation of the same discourse, but not that they influence each other and reinforce the opinions of their siblings. In other words, users who refer to the same news sources may never meet on the platform. In fact, the information about who interacts with whom is not used to detect user NECs.

As mentioned in the Introduction section, users in an echo chamber are users who share a common discourse, are exposed to the same news sources, and are exposed to the same opinions. Being exposed to the same opinions, translated to Twitter, means that they retweet each other. In this sense, if users in the same user NEC form a (weakly) connected component in the same DiCo-induced subgraph of the retweet network (i.e. if there is a flow of influence in the retweet network that is restricted to nodes in the same discursive community), they form an echo chamber.

The analysis of the weakly connected component shows that 92 users do not belong to it. This leaves 473 users trapped in echo chambers. In particular, all users in user NECs 8, 9, and 10 did not retweet others in the same user NEC on the topic under analysis. Regarding the other user NECs, we observe that for each of them, most of the nodes form echo chambers. In the following, echo chambers inherit the ID of their user NEC. Some echo chambers are relatively large: for example, those induced by user NECs 1 and 2 contain more than 100 nodes.

To study how much users in echo chambers are connected, we use the undirected clustering coefficient: ignoring the direction of the edges, it captures the observed frequency of interactions between the neighbors of each node (16).

**Fig. 4.**
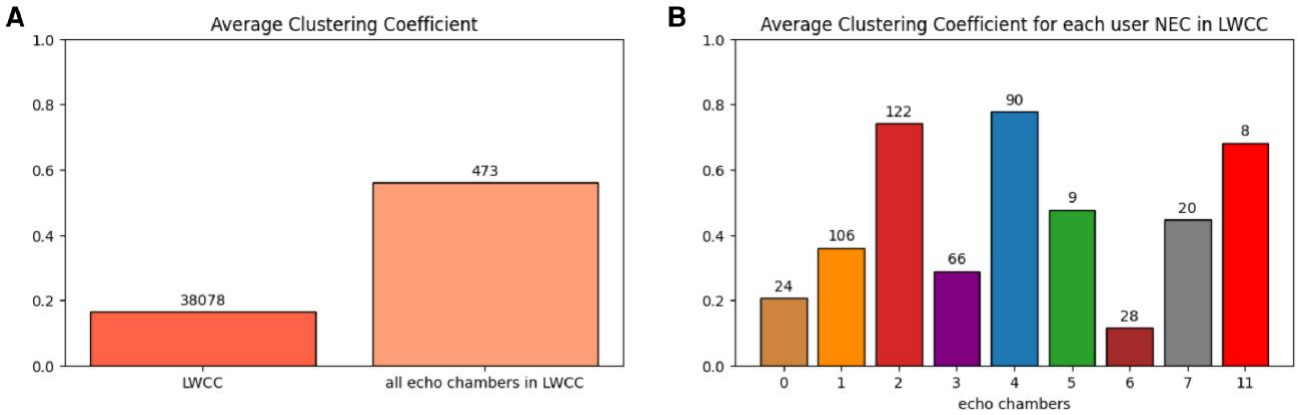
A) Average clustering coefficient measured on the LWCC of the retweet network restricted to users of Fdi-l-media and measured on all users belonging to echo chambers. B) Average clustering coefficient calculated on each echo chamber. Each echo chamber inherits the ID and the color from its user NEC. The number of users in the echo chamber is shown at the top of each bar.

We compare the clustering coefficients of the echo chambers with the one measured on the Largest Weakly Connected Component (LWCC) of the retweet network restricted to users in the Fdi-l-media DiCo. In this way, we have a benchmark that captures the main contribution to the discourse to which the echo chambers belong. The clustering coefficient associated with users in echo chambers is more than three times as high as that for other users within the LWCC [0.56 compared to 0.16, Fig. 4(A)]. We then examine the average clustering coefficient within each echo chamber. Figure 4 shows that the average clustering coefficients of echo chambers 2, 4, and 11 are greater than 0.6.

High values of the clustering coefficient imply that accounts are highly connected and frequently retweet each other. Therefore, we can conclude that their endorsement activity contributes to the reinforcement of their opinions. Such a conclusion is confirmed by a manual examination of the content shared by users in echo chambers after almost 2 years. At the time of the data collection, the opinions of the users were strongly against the Covid-19 vaccination. After 2 years, the positions of the users there still adhere to conspiracy theories and have become particularly extreme.

To provide a concrete example, we focus on the content shared in echo chamber 4. In practice, we first manually extract the main narratives from the news shared within echo chamber 4, focusing on the users with the highest number of followers at the time of data collection. Then, still focusing on the users with the highest number of followers, we analyze whether there are signals of these narratives in their most recent posts (as of 2023 June 7) and which narratives they currently support. In echo chamber 4, there are about 1.7k unique news that have been shared about 7.3k times in total. First, we exclude the news with connection errors at the time of this analysis (1k shares) and those that have been shared less than 10 times. Then, we analyze the resulting news narratives, which amount to ∼3.3k shares and 146 unique URLs from 51 different domains. By classifying only these 146 news stories, we cover about ∼45% of the total URL traffic within echo chamber 4. Table 2 shows the narratives’ distribution and their descriptions: the main eight narratives are all against vaccination and government regulations.

**Table 2.** Narratives’ descriptions in echo chamber 4.

ID	Narrative	#url supporting the narrative
1	Protests against the Covid-19 green pass	673
2	Comments on presumed deaths after vaccine	536
3	comments on presumed injuries after vaccine	510
4	Statements made by politicians against vaccinations	495
5	Against mandatory vaccination	441
	News about VIPs rejecting the vaccine.	
	Ineffectiveness of vaccines.	
6	Mattarella incites to hatred no-vax. Experts reject the third dose	308
7	(Manipulated) data about vaccine hazard versus efficacy	196
	and hospitalizations or infections despite vaccination	
8	COVID-19 vaccines are still too experimental	126
	Police forces were not vaccinated. Support to views of no-vax doctors.	
	VIPs and high-ups pretend to be vaccinated, but actually are not	

Table 3 shows the narratives supported by the users in echo chamber 4 with the most followers, almost 2 years after data collection (2023 June 7). Users hold extreme views on current controversial issues such as the war in Ukraine, migrants, and LGBT issues. Remarkably, conspiracy theories about vaccines are still present in their narratives.

**Table 3.** Main narratives supported in recent posts (as of 2023 June 7) by users in echo chamber 4 with the most followers.

User	Followers	Supported narratives
user_1	36,926	No-migrants, no-vax, anti-EU
user_2	6,929	Pro-Russia, no-vax, no-LGBT
user_3	6,335	Pro-Russia, no-migrants, anti-EU, conspiracy theories
user_4	4,164	No-vax, no-migrants, pro-Russian
user_5	3,117	no-vax
user_6	2,668	Pro-Russian, against the Italian government, no-vax
user_7	2,641	suspended
user_8	2,448	Conspiracy theories, no-vax, no-LGBT, against the Italian government, anti-EU
user_9	2,355	Religious posts, no-green pass, no-vax
user_10	2,316	Against Italian government, no-vax

Users are anonymized.

### Echo chambers, their role in the common discourse and the plague of misinformation

**Fig. 5.**
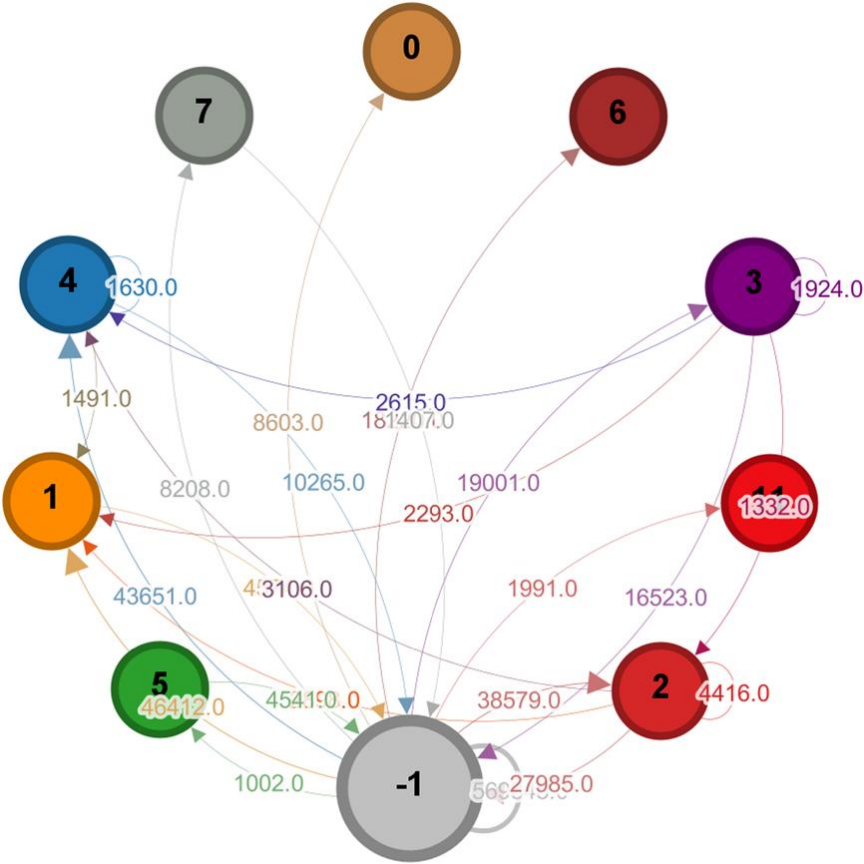
Retweet network for Fdi-l-media DiCo, aggregated with respect to echo chambers. Node −1 represents users who do not belong to an echo chamber. Edges indicate the number of retweets between different user groups; weights less than 1k have been filtered out.

Figure 5 shows the flow of retweets within an echo chamber and between different echo chambers.

Node −1 represents all nodes in the DiCo that are not part of an echo chamber, and an arrow indicates that tweets published by the source group are retweeted by a certain number of users in the target group. Self-loops represent retweet activity within the same group. The values on the edges indicate the number of retweets associated with each interaction. Although the echo chambers are composed of a small number of users (on the order of $10^{2}$, compared to the total number of DiCo users, on the order of $10^{4}$), they contribute significantly to the DiCo’s retweet activity. Echo chambers are involved in generating about 288k retweets, while users not in echo chambers generate about 569k retweets. More specifically, echo chambers 2 and 3 are mainly composed of popular users (in terms of received retweets), while others are mainly composed of retweeting users (0, 1, 4).

To quantify the presence of misinformation in echo chambers, we have tagged URLs in our dataset that point to news sites. The labels are those that the NewsGuard journalistic organization has assigned to online media outlets.^a^ The use of the labels has been licensed to the authors of this article. More details about the reputability measure implemented in the present manuscript can be found in Section S3.

**Fig. 6.**
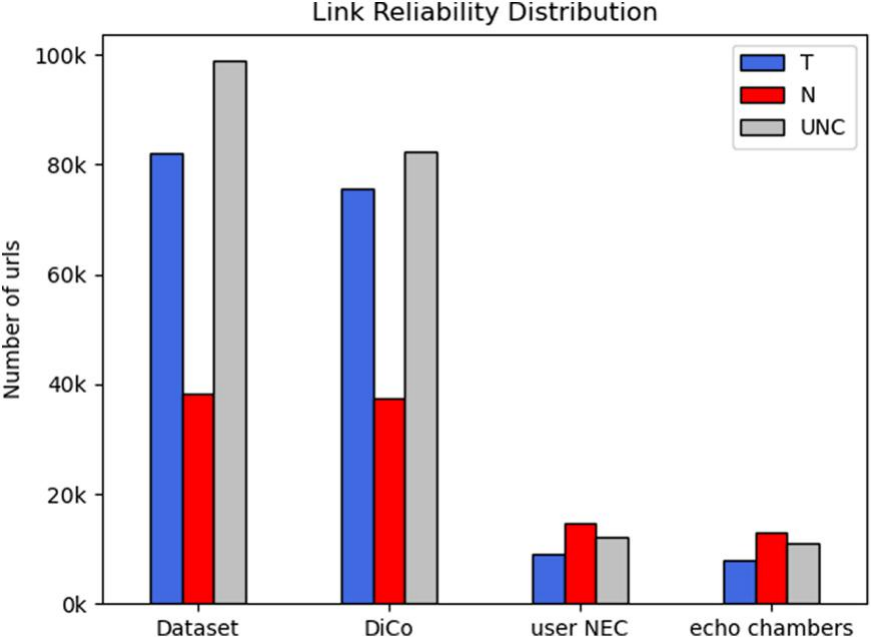
Number of distinct URLs pointing to news publishers tagged as “Trustworthy” (T), “Not trustworthy” (N), or “Unclassified” (UNC) for the entire dataset and for each type of users’ community (DiCos, user NECs, echo chambers.)

Figure 6 shows the number of URLs pointing to news from publishers that NewsGuard classifies as “Trustworthy” (T), “Not Trustworthy” (N), and “Unclassified” (UNC) for the entire dataset and for each type of user community. If the same URL is shared multiple times by users in the same group, this multiplicity is taken into account in the analysis.

The first observation is that the differences between user NECs and echo chambers are negligible. Second, DiCos cover almost the entire volume of both T and N traffic. Remarkably, while the ratio between untrusted and trusted URLs is around 0.5 for the entire dataset, the ratio is almost reversed for echo chambers: the frequency of N news sources is almost twice that of T news sources.

Section S4 will show even more alarming results regarding the spread of disinformation in echo chambers. We do not show these results in the main text due to space limitations, but i) the probability that a link shared by a user in an echo chamber refers to an untrustworthy news source is 0.377, compared to 0.129 for users outside echo chambers; ii) the probability that a link shared by a user in an echo chamber refers to a trustworthy news source is 0.232, compared to 0.379 for users outside echo chambers.

## Conclusion

In this article, we propose a novel unbiased method to detect echo chambers. The method is mainly based on two observations. First, echo chambers form when users interact with others who share similar opinions and refer to the same news. Second, a proper null model should be implemented to detect a true signal. This necessity has recently been highlighted in the literature on online social networks and has been shown to be particularly important for the detection of nontrivial phenomena (6–8, 17–19). Our echo chamber detection method is based on the validation of observed structures by comparison with a proper maximum entropy null model; the maximization of entropy guarantees the unbiased nature of the benchmark.

We tested our procedure on a dataset containing the Italian Twitter debate on Covid-19 vaccination: we found that our procedure detects a low presence of echo chambers (just under 0.35% of all users in our dataset belong to an echo chamber). All the echo chambers we detected are part of the same discursive community, i.e. a community of users with similar political positions. Even if their dimension in terms of the number of users is limited, their impact on the shared discourse is remarkable: echo chambers are responsible for almost a third of the retweets in their discursive communities.

The methodology can be extended to other online social networks. In fact, it is based on (i) the analysis of the activity of accounts that share URLs to news sources and (ii) the detection of discursive communities. While the extension of the former to other online social networks is straightforward, the latter may be more problematic: in the present case, we used the activity of verified users, who are among the main content creators in Twitter (6), but not all social platforms have such certification. Nevertheless, when analyzing other platforms, we can still focus on users who are particularly active in creating new content, such as influential users as defined in (20).

Not unlike other studies, our study has some limitations, which we believe do not affect our final conclusions. First, it may be argued that the validation procedure is quite strict: the validation of multiple *P*-values leads to the validation of extreme events. While this is true, it is the only way to eliminate random noise from the system and analyze the true signal (see Sections S5 and S6 for more details). Finally, the main idea of echo chambers is that users follow accounts with similar ideas, while in the present study only the retweet network is used, not the information about friendships. Still, the retweet network captures the effective interactions with interesting content as perceived by different users, whether it comes from friends or is suggested by the platform itself: focusing only on friendship will not fully capture the effect of the platform’s recommendation algorithm.

## Methods

### Network analysis methods

Recently, De Clerck et al. stressed the importance of using proper statistical benchmarks for the analyses of Online Social Networks (18, 19): in fact, such systems are affected by strong noise and detecting genuine signals is fundamental in order to drive the proper conclusions. In fact, our procedure for the detection of echo chambers is based on the statistical validation of different co-occurrence networks. Co-occurrences are implicitly based on a bipartite structure: if we count, for instance, the number of URLs that have been shared by both a pair of users, we are implicitly projecting the information contained in a bipartite network in which layers represent users and URLs on the layer of users. Therefore, including the bipartite information in the analysis of the observed co-occurrences provides a more accurate benchmark.

A general framework for providing unbiased benchmarks for the analysis of complex networks was recently proposed in the literature (5), inspired by the derivation of Statistical Physics from Information Theory by Jaynes (21). The main idea is to first create an *ensemble* of all graphs having the same number of nodes as in real systems. We can then define the Shannon entropy associated with the ensemble: in order to have a maximally random benchmark, we maximize the Shannon entropy, constraining some defining quantities about the system. In this sense, by comparing the real network with our null model, all observations that cannot be explained by the constraints can be captured. Constraints can be global, as the total number of links, or local, as the degree sequence, i.e. the number of connections per node.

In the following, we will first introduce the Bipartite Configuration Model (*BiCM*, (22)), i.e. the application of the procedure described above to bipartite networks in which the degree sequences are the constraints. Then we will describe the validation procedure for co-occurrences, proposed in Ref. (23). Both the BiCM and the validation procedure used in the present manuscript were performed using the bicm^b^ python module included in NEMtropy;^c^ the methods used to solve BiCM system of equations implemented in NEMtropy and bicm can be found in Ref. (24).

#### Formalism

In a bipartite network, nodes are divided into two sets, called *layers* and links exist only between nodes belonging to different layers. Given a bipartite network $G_{\mathrm{Bi}}$, let us call its layers ⊤ and ⊥, respectively, and $N_{⊤}$ and $N_{⊥}$ their dimensions. Then, a bipartite binary network is completely described by its biadjacency matrix B, i.e. a $N_{⊤}\times N_{⊥}$ rectangular matrix whose generic entry $b_{i\alpha }$ is either 1 or 0 if there exists a link connecting node i∈⊤ and α∈⊥ or not. The degree of a generic node i∈⊤ (α∈⊥) is simply $k_{i}=\sum_{\alpha \in ⊥}b_{i\alpha }$ ($h_{\alpha }=\sum_{i\in ⊤}b_{i\alpha }$). In the following, quantities related to real networks will be indicated with an asterisk *.

#### Bipartite configuration model

Let us call $G_{\mathrm{Bi}}$ the ensemble of graphs of the BiCM in which each representative graph $G_{\mathrm{Bi}}\in G_{\mathrm{Bi}}$ is a $N_{⊤}^{*}\times N_{⊥}^{*}$ bipartite network.^d^ We define the Shannon entropy associated with the system as


(1) $$S=-\sum_{G_{\mathrm{Bi}}\in G_{\mathrm{Bi}}}P(G_{\mathrm{Bi}})\ln\;P(G_{\mathrm{Bi}}).$$

We can perform a constrained maximization of the Shannon entropy using the methods of Lagrangian multipliers, the constraints being the degree sequences of both layers, i.e. $k_{i},\;\forall \;i\in ⊤,$ and $h_{\alpha },\;\forall \;\alpha \in ⊥$. In this way, we will achieve a benchmark that is maximally random, but, in which the average degree sequences are equal to the ones observed in the real system. Therefore, by observing deviations from the null model we will detect all structures of the real system that cannot be simply explained by the constraints. Such a procedure can be achieved through the maximization of the function $S^{\prime }$ defined as


$$\begin{array}{r} S^{\prime }=S+\beta \left(1-\sum_{G_{\mathrm{Bi}}\in G_{\mathrm{Bi}}}P(G_{\mathrm{Bi}})\right)+\sum_{i\in ⊤}\theta_{i}(k_{i}-\langle k_{i}\rangle )+\sum_{\alpha \in ⊥}\eta_{\alpha }(h_{\alpha }-\langle h_{\alpha }\rangle ), \end{array}$$

where *S* is the Shannon entropy defined in Eq. 1 and *β*, $\theta_{i}$, and $\eta_{\alpha }$ are the Lagrangian multipliers associated, respectively, to the normalization of the probability, to the degree sequence on layer ⊤ and to the degree sequence on layer ⊥. The maximization of $S^{\prime }$ returns a probability per graph that can be written in terms of independent probabilities per link (25):


(2) $$P(G_{\mathrm{Bi}})=\prod_{i,\alpha }\frac{e^{-(\theta_{i}+\eta_{\alpha })b_{i\alpha }}}{1+e^{-(\theta_{i}+\eta_{\alpha })}}=\prod_{i,\alpha }p_{i\alpha }^{b_{i\alpha }}(1-p_{i\alpha }{)}^{1-b_{i\alpha }}.$$

Equation 2 is just formal since we do not know the numerical value of Lagrangian multipliers $\theta_{i}$ and $\eta_{\alpha }$. This can be obtained through the maximization of the likelihood of observing the real system (26, 27). It can be shown that maximizing the likelihood is equivalent to set:


$$\left\{ \begin{array}{c} \langle k_{i}\rangle =k_{i}^{*}\\ \langle h_{\alpha }\rangle =h_{\alpha }^{*}. \end{array}\right.$$

In Section S5, the interested reader can find a detailed description of how to use the Bipartite Configuration Model as a statistical benchmark to validate the co-occurrences observed in the real network.

### Discursive communities

As stated above and described in detail in Section S5, the BiCM described above can be used as a statistical benchmark to highlight groups of users contributing to the formation of the same discourse. On Twitter, this translates to groups of users endorsing similar content. In Ref. (6) a procedure was proposed, later refined in Refs. (8, 9, 17). The rationale is to consider who are the creators of content and how to capture similarities among them. It has been observed in several studies that verified accounts, i.e. the ones for which the Twitter platform checked the identity of their owners—at least in the pre-Musk era—are strong creators of content (6, 17). It is possible, then, to infer how similar they are perceived by the “general” public of unverified users by using a bipartite representation: if verified and unverified users are the two layers of a bipartite network in which the (undirected) links represent retweets,^e^ we can validate the projection on the layer of verified users. In this way, we will detect nontrivial similarities in the common audience of unverified users: otherwise stated, if a couple of verified users are retweeted by the same (nonverified) users, they are probably sharing similar positions. In the monopartite validated projection of verified users, communities were detected using an optimized version of Louvain algorithm (15): since Louvain is known to be node-order dependent (28), the order of the nodes is shuffled 1,000 and the configuration displaying the greatest value of the modularity is chosen. The labels of the communities found through the community detection are then propagated in the retweet network: in fact, it is an old result that Twitter users endorse content created by others much more with retweets than with mentions (10–12). Since in many cases, some strong creators of content are not verified (and therefore run the risk of not getting a label), we run the label propagation algorithm on the undirected version of the retweet network: the rationale is that not only the sources give an indication of the user orientation but also her audience. Otherwise stated, if the majority of a user audience has a clear orientation, it is presumable that the considered user also has the same one. For propagating labels, we implemented the procedure proposed in Ref. (13). This algorithm assigns the unverified user the label associated with the majority of its neighbors in the retweet network. If an unverified user has no verified users as direct neighbors, it will be assigned the label associated with the majority of unverified neighbors that have already been labeled. This continues iteratively until it converges.

The interested reader can find in Section S6 a comparison between DiCos obtained using validated or nonvalidated projections. In summary, it has been observed that politicians are particularly clustered in the validated network (6–9, 17, 29–31). Therefore, detecting community therein is particularly efficient in finding discursive communities about political subjects. On the contrary, the discursive communities calculated on the nonvalidated projection are much noisier.

### News engagement communities

#### URL manipulation

To detect similarities in users’ endorsement of pieces of news, we first need to pre-process the URLs contained in the various tweets. Sharing a compact version of a URL allows for the sharing of long URLs in tweets while maintaining the maximum character limit. For our analyses, we translated all shortened links into their original long versions. This enabled us to (i) read the top-level domain of the news source to assign a nutrition label using NewsGuard and (ii) use the long links as unique identifiers for each shared news item in our network models.

#### NEC communities

In order to find users sharing similar “information diets”, i.e. engaging with the same URLs, we used the same approach as in Ref. (14). We first represented users sharing (either via tweets or retweets) URLs as a bipartite network of users and URLs. Then, we projected the information contained therein on the layer of users and finally validated the projection using the procedure described above. As mentioned in Section 2.3, the fraction of validated nodes, in this case, is extremely limited, i.e. nearly 1.71%, signaling that most of the users’ endorsement to URLs (and so pieces of news) is compatible with the random noise. Again, in order to find communities of users in the validated projection network, the reshuffled version of Louvain was used.

## Literature review

The detection of echo chambers has generally been approached in the literature by starting with online content whose nature is known a priori. By analyzing the social accounts that interact with specific content, e.g. through likes, shares, retweets, and comments, it has been shown how information related to specific narratives attracts particular communities. As an example, the work in (1), by Del Vicario et al., focuses on public Facebook pages divided into two groups: conspiracy theories and science news (conspiracy theories are “the pages that disseminate alternative, controversial information, often without supporting evidence” (1)). The results show that users are divided into homogeneous clusters: by analyzing the accounts that share news about science and conspiracies, they are bound by friendship ties in the network. To quote the authors: “different contents generate different echo chambers, characterized by the high level of homogeneity inside them”.

Homogeneity is not only about friendship but also about emotional approach and response to debunking attempts. Zollo et al., in (32), note how users polarized on conspiracies express more negative feelings in their comments than users polarized on science news. Work in (4) confirms how the echo chamber paradigm goes hand in hand with the phenomenon of confirmation bias—the tendency of users to seek out, favor, and interpret information in line with their thoughts (33, 34), while ignoring or downplaying evidence that contradicts their beliefs: interactions with debunking posts (i.e. posts that provide fact-checked information on specific topics) are overwhelmingly from science-biased or nonbiased users.

Interestingly for the purposes of this article, other studies have instead analyzed the dynamics of information exposure by looking at the news URLs present in the posts. This is the case, for example, of the work by Weaver et al. (35), which constructs the network of densely connected news articles. It starts from the number of news URLs shared by each user to arrive at the weighted network of news URLs, where the weights between two URLs indicate how many users have re-shared the URLs. Leveraging a state-of-the-art community detection algorithm, communities of co-shared news items are found, distinct in terms of political leaning (i.e. left-leaning and right-leaning). Guarino et al., in (14), consider public Facebook pages without knowing a priori the kind/quality/reputability of their content. Focusing on the activity of users sharing links to pieces of online news, the authors construct the bipartite network of users/shared URLs and apply the Bipartite Configuration Model (BiCM) to project the bipartite network at the two levels, the user level and the URL level.

## Supplementary Material

pgae177_Supplementary_Data

## Data Availability

The X/Twitter dataset is available at https://doi.org/10.6084/m9.figshare.25460962.v1 The dataset is completely anonymized, in agreement with X/Twitter’s policies, https://twitter.com/en/privacy. The association between the trustworthiness labels and the news sources that supports the findings of this study is proprietary Newsguard data.

## Notes

a
https://www.newsguardtech.com/solutions/newsguard/

b
https://pypi.org/project/bicm/

c
https://pypi.org/project/NEMtropy/

d Since all networks will have the same number of nodes in each layer, asterisks will fall only from $N_{⊤}^{*}$ and $N_{⊥}^{*}$.

e In this representation, a link connecting a verified users *v* with a nonverified *u* is present if at least one of them has retweeted the other at least once. Due to the strong production activity of verified accounts, nearly all of the links are from the layer of verified users to the opposite one.

## Previous presentation

Some of the results were presented at NetSci 2023, Wien, on 2023 July 14th.

## Preprints

An earlier version of this work is available at https://doi.org/10.48550/arXiv.2308.01750.
