# Supplementary material for: Entropy-based detection of Twitter echo chambers
Source: PNAS Nexus. 2024 Apr 25;3(5):pgae177. doi: 10.1093/pnasnexus/pgae177 (PMC11086943; doi:10.1093/pnasnexus/pgae177)
Supplement: pgae177_Supplementary_Data [file pgae177_supplementary_data.pdf]

Supplementary Information for Entropy-based detection of Twitter echo chambers

Manuel Pratelli[a,d], Fabio Saracco[b,c,a,*], and Marinella Petrocchi[d,a]

[a]IMT School for Advanced Studies, Piazza San Francesco 19, 55100 - Lucca (Italy)
[b]'Enrico Fermi' Research Center (CREF), Via Panisperna 89A, 00184 - Rome, Italy
[c]Institute for Applied Computing "Mauro Picone", CNR, Via dei Taurini 19, 00185 - Rome (Italy)
[d]Istituto di Informatica e Telematica, CNR, via G. Moruzzi 1, 56124 - Pisa (Italy)
[*]fabio.saracco@cref.it

1 Keywords for data collection

**Keywords**	**English meaning**
vax, vaccino, vaccini, vaccinarsi	Variants of the word 'vaccination'
novax	A person against vaccination
Astrazeneca, Pfizer-BioNTech, Moderna, Sputnik	Covid-19 vaccines
greenpass	The certificate of vaccination or of recover from the disease

Table S1: **Keywords used for collecting tweets about the Twitter debate on the Covid-19 vaccination campaign.** Keywords have been searched in Italian, English meanings on the right.

2 News Engagement Communities of URLs

Similar to what was done in the main text to find validated communities of users, it is possible to analyze the ties between users and the URLs present in their tweets and retweets to find validated communities of URLs. Again, the procedure involves a comparison between the observations and an entropy-based benchmark: if two URLs appear in the tweets (or retweets) of the same users significantly more than the benchmark, these URLs pass the validation procedure: We can thus identify groups of URLs shared by the same users. URL communities that pass the validation are called *news engagement communities* of URLs,

Table S2 summarizes the breakdown of URLs into URL NECs: Only 22% of all URLs are validated by our procedure.

Table S2: **URLs in URL NEC.** Although validated URLs represent a limited minority of all URLs in the dataset, their percentage is greater than their user analogues (i.e. 22% vs. 2%).

Comm. ID	No. URL
Non-validated	179,175
Validated	51,504

Table S3: **Statistics for URL NECs.** While community 4 is by far the largest, there are 6 other communities with more than 1000 URLs.

Comm. ID	No. Users	verified	distinct URL	sources	No.URL
4	7422	16	223	71	38731
1	674	1	79	9	2234
6	876	0	87	5	2019
11	1064	4	21	4	1681
7	521	0	64	6	1613
10	65	1	27	9	1557
9	584	1	58	1	1238
5	311	0	23	6	562
12	175	1	28	4	408
3	101	1	79	1	393
0	161	0	55	1	365
8	149	0	25	1	308
13	253	0	3	3	304
14	42	0	4	2	59
2	23	0	3	3	32

More details can be found in Table S3, which shows some information about the different URL NECs. URL NEC 4 is the largest in terms of both size (consisting of 223 nodes) and impact on the overall dataset, as measured by the number of shares ($\sim$ 39k shares). The remaining URL NECs can be distinguished based on the order of magnitude of the shares: we have 6 communities whose URLs were shared thousands of times, and other communities whose URLs were shared hundreds or dozens of times. Remarkably, in all but 4 of the URL NECs, the number of different sources is quite limited (where source means the online news outlet that published the news to which the URL points).

To get a finer description of URL NECs, we examine the frequency of untrustworthy news sources in them. For each URL pointing to a news article, we consider the corresponding second-level domain[1], which refers to the name directly to the left of .com, .net, and other top-level domains (such as `nytimes.com` and `latimes.com`). We then associate the domains with the publishers, annotating the former with the reputation labels provided for the latter by the NewsGuard site (`https://www.newsguardtech.com/`). In this sense, the trustworthiness of a URL is inherited from the trustworthiness of its domain/publisher, i.e. a news item is considered more or less trustworthy depending on the trustworthiness of its publisher. According to the NewsGuard classification, the labels T ('Trustworthy'), N ('Not trustworthy') and UNC ('Unclassified') stand for the level of trustworthiness of the publisher. For more details on how the information is processed by NewsGuard, see Section 3.

The first observation is that URL NECs are a receptacle of untrustworthy sources, see Fig. S1. With respect to the total number of distinct URLs in our dataset, URL NECs capture less than half of the trustworthy ones, but almost all of the untrustworthy ones.

Fig. S2 pictorially shows the network of URL NECs, as it emerges from the data. The different communities show a strong homogeneity in the trustworthiness of their sources.

To investigate more deeply the level of homogeneity of the single community in terms of the trustworthiness label of URLs within them, we consider the frequency of trustworthy and untrustworthy sources of URLs therein. For the i-community of URL NECs, if R is the trustworthiness value (either T or N), we define $purity_R(\text{URL NEC}_i)$ the frequency of URLs from R domains, i.e.

$$purity_R(\text{URL NEC}_i) = \frac{|U_i^R|}{|U_i|}, \tag{S.1}$$

[1]`https://en.wikipedia.org/wiki/Domain_name`

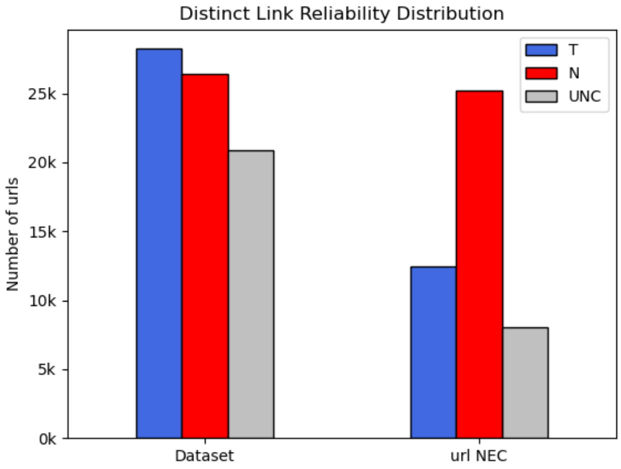

Figure S1: **Number of distinct URLs pointing to publishers tagged as 'Trustworthy' (T), 'Not trustworthy' (N), or 'Unclassified' (UNC).** URL NECs capture almost all non-trusted unique URLs in our dataset.

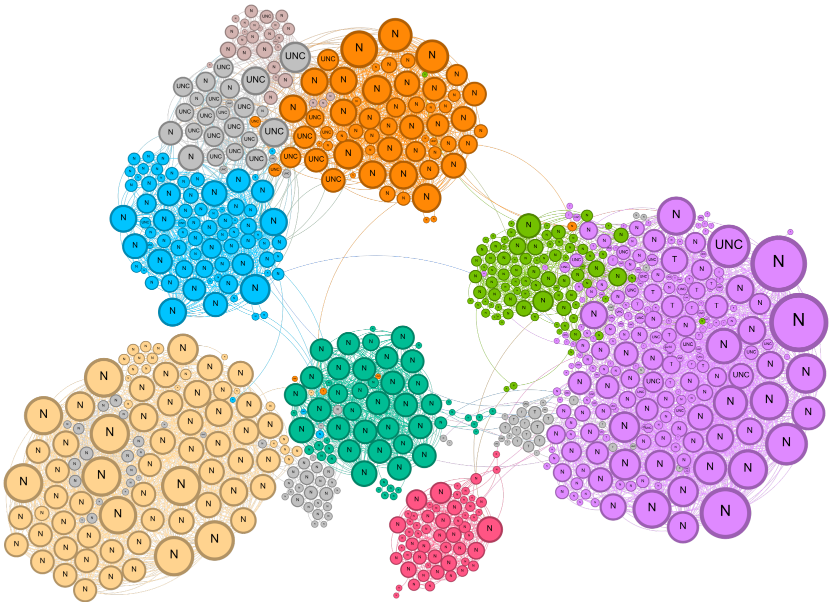

Figure S2: **Network representation of URL NECs.** The labels on the nodes represent the trustworthiness of the domain of the URL as labeled by NewsGuard (T for 'Trustworthy', N for 'Not trustworthy', UNC for 'Unclassified' sources). Each community shows a strong homogeneity in the trustworthiness label.

where $U_i = \{URL_1, \ldots, URL_n\}$ is the set of all the URLs in the i-community and $U_i^R \subseteq U_i$ is the subset of U_i that contains only URLs with trustworthiness R. The purity defined in Eq. S.1 can be interpreted as the probability of extracting an R-reputable URL in the i-th URL NEC. If m is the

number of different URL NECs, we can define $purity_R(\cup_i \text{URL NEC}_i)$ as the frequency of URLs from R domains in all URL NECs:

$$purity_R(\cup_i \text{URL NEC}_i) = \frac{\sum_{i=1}^{m} |U_i^R|}{\sum_{i=1}^{m} |U_i|} \tag{S.2}$$

To have a benchmark for the purity of URL NECs, we also consider a purity measure for URLs that do not belong to any community:

$$purity_R(\overline{\cup_i \text{URL NEC}_i}) = \frac{|U_{-1}^R|}{|U_{-1}|}, \tag{S.3}$$

where the set of URLs that do not belong to any community is denoted as U_{-1}.

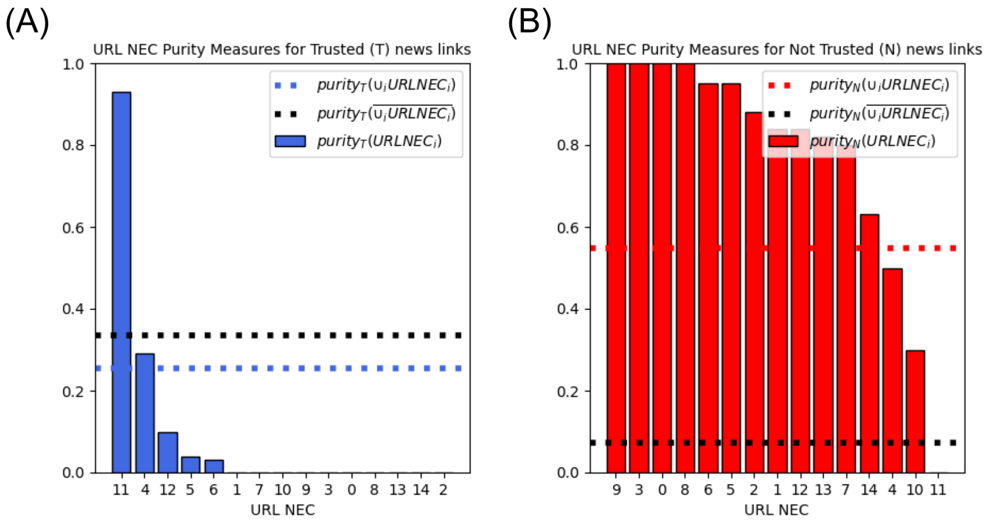

Figure S3: **Purity levels of URL NECs.** In panel (A) trustworthy URLs, in panel (B) untrustworthy ones. While the T purities of the individual communities are particularly low (less than 0.2 in most cases), the analog N purities are greater than 0.6 for most of the URL NECs.

Fig. S3 shows the homogeneity of URL NEC communities concerning trustworthy (T, panel (A)) and untrustworthy (N, panel (B)) news sources. On the x-axis there are the URL NEC communities, denoted by their ids, while the y-axis reports the purity of each community. The blue dotted line indicates $purity_R(\cup_i \text{URL NEC}_i)$, the black dotted line indicates $purity_R(\overline{\cup_i \text{URL NEC}_i})$. Focusing on the $purity_R(\cup_i \text{URL NEC}_i)$ lines, on average URL NECs have higher N purity URLs (~ 0.548) compared to T URLs (0.254). Such a result suggests that URLs belonging to URL NECs represent niches of misinformation sources, and it is corroborated by the observation that most of URL NECs refer to a limited number of different sources (see Table S3).

3 Article's reputability measure (NewsGuard)

One of the aims of the work is to characterize the variety of domains circulating within the dataset, both in terms of type (e.g., news site, marketplace, social platform, etc.) and transparency and credibility (only in the case of news sites). In this paper, we refer to domains as the 'second-level domain' names[2],

[2] https://en.wikipedia.org/wiki/Domain_name

i.e., the names directly to the left of .com, .net, and any other top-level domains. For instance, we consider domains nytimes.com, guardian.com, corriere.it.

The domains have been tagged according to their degree of credibility and transparency, as indicated by fact-checking website NewsGuard (https://www.newsguardtech.com/). The NewsGuard initiative was born from the joint effort of journalists and software developers, aiming at evaluating news sites according to criteria concerning credibility and transparency. For evaluating the credibility level of a source of information, NewsGuard metrics consider, e.g., whether the news source regularly publishes false news, whether it distinguishes between facts and opinions, or whether it does not correct a wrongly reported news. For transparency, instead, NewsGuard evaluation takes into account, e.g., whether owners, founders or authors of the news source are publicly known, or whether advertisements are easily recognizable[3].

label	description
T	Trustworthy news source
N	Not trustworthy news source
P	Platform (e.g., reddit.com, twitter.com, facebook.com)
S	Satire
UNC	Unclassified source

Table S4: Tags for domain labelling. Tags are inherited from NewsGuard. The UNC tag indicates that NewsGuard did not tag that domain.

Table S4 shows the tags associated with domains. In the manuscript we shall be interested in quantifying the reliability of news sources that were publishing during the period of interest. Thus, we will not consider those sources corresponding to social networks (tag P). Also, we will not consider satiric news (tag S). Tags T and N in Table S4 are used only for news sites, be they newspapers, magazines, TV or radio social channels, and they stand for 'Trustworthy' and 'Not trustworthy', respectively.

4 Exposure of users to misinformation in echo chambers

To provide a finer characterization of users' exposure to misinformation in echo chambers, we 'recycle' the purity definition of Section 2, with one crucial difference: there, the purity measure was applied to different sets of URLs from time to time; here, we apply it to all messages shared by different sets of users. Thus, in the present case, if a URL has been shared multiple times, we consider the repetitions. The rationale for this is to characterize echo chambers in terms of the extent to which links to news stories from untrustworthy news publishers circulate within them. If $|\mathrm{EC}_i(\mathrm{URL})|$ and $|\mathrm{EC}_i(\mathrm{URL}; R)|$ count, respectively, the number of messages containing a URL and a R-reputable URL shared by users in echo chamber i, with a little abuse of notation we can define a purity for echo chamber as

$$purity_R(\mathrm{EC}_i) = \frac{|\mathrm{EC}_i(\mathrm{URL}; R)|}{|\mathrm{EC}_i(\mathrm{URL})|}. \tag{S.4}$$

Analogously to what was done in Subsection 2, we can define $purity_R(\cup_i \mathrm{EC}_i)$ and $purity_R(\overline{\cup_i \mathrm{EC}_i})$, respectively for all users in echo chambers and for all users outside echo chambers. The results of the analysis is reported in Fig. S4: on the x-axis there are the echo chambers denoted by their ids, on the y-axis the purity values. In panel (A), purities are related to trustworthy URLs. In panel (B), purities are related to untrustworthy URLs. The blue dotted line indicates $purity_R(\cup_i \mathrm{EC}_i)$, the black dotted line indicates $purity_R(\overline{\cup_i \mathrm{EC}_i})$.

[3]Details on the news site evaluation starting from the estimate of the assessment criteria are available at: https://www.newsguardtech.com/ratings/rating-process-criteria/.

Focusing on the $purity_R(\cup_i \mathrm{EC}_i)$ lines, echo chambers on average have a higher purity with respect to untrustworthy URLs (~ 0.377) compared to trustworthy ones (~ 0.232). In other words, when a user posts a message containing a URL in an echo chamber, the probability that it points to an untrustworthy news source is close to 0.4; for some echo chambers, this probability is even much higher than this. As in the case of the purity for URL NECs, if we compare the $purity_R(\cup_i \mathrm{EC}_i)$ values against $purity_R(\overline{\cup_i \mathrm{EC}_i})$, there is a trend reversal in passing from T to N: the $purity_T(\overline{\cup_i \mathrm{EC}_i})$ value is greater than its counterpart in the echo chamber while $purity_N(\overline{\cup_i \mathrm{EC}_i})$ is lower than the value measured in echo chambers. This finding is worrisome because users in echo chambers are particularly polarized and committed, basing their beliefs on low-quality news. However, it is important to remember that the formation of echo chambers, while alarming in itself, is generally unrelated to the quality of news sources.

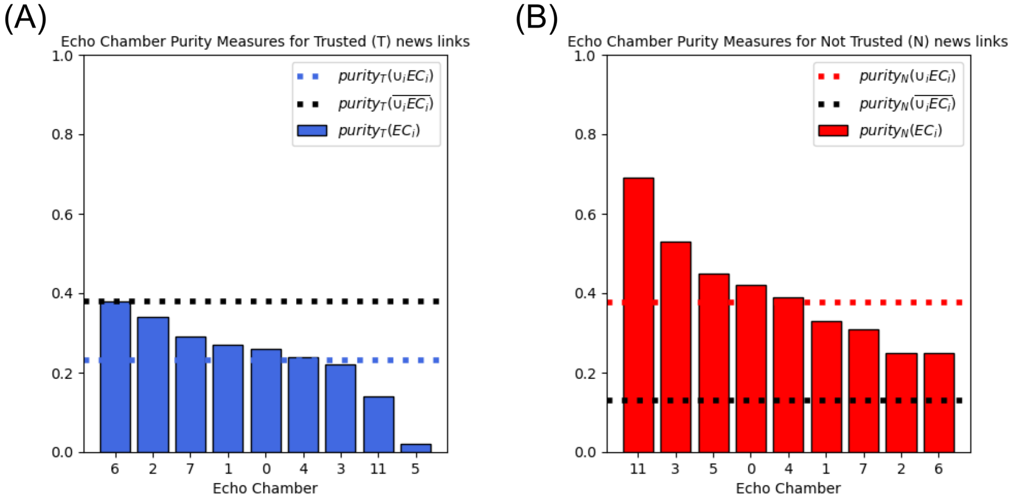

Figure S4: **Purity levels of echo chambers.** In panel (A) trustworthy URLs, in panel (B) not trustworthy URLs. While the $purity_T(\overline{\cup_i \mathrm{EC}_i})$ value is greater than its counterpart in the echo chamber, $purity_N(\overline{\cup_i \mathrm{EC}_i})$ is lower than the value measured in echo chambers.

5 Validated projection of bipartite networks

The BiCM null model introduced in Subsection 4.1.2 of the main text can be used to validate the co-occurrence network defined from a bipartite one. Consider two nodes $i, j \in \top$: the number of co-occurrences between them is

$$V^{ij} = \sum_{\alpha \in \perp} V^{ij}_\alpha = \sum_{\alpha \in \perp} b_{i\alpha} b_{j\alpha}. \tag{S.5}$$

As mentioned in the subsection above, the probability of observing a graph G_{Bi} is factorised in terms of probabilities of the existence of a single link. Therefore the probability that both nodes i, j link a single node $\alpha \in \perp$ is simply

$$P(V^{ij}_\alpha) = p_{i\alpha} p_{j\alpha},$$

where V^{ij}_α is defined in Eq. S.5. In general, given node $i \in \top$, all $p_{i\alpha}$ are different, depending on the degree h_α. In this sense, the BiCM probability distribution of V^{ij} is the generalization of the binomial distribution in which each event V^{ij}_α has a different probability. Such a distribution is known in the literature with the name of Poisson-Binomial distribution. For each observed co-occurrence, we can

then calculate its p-value [12].

Finally, all p-values are validated using a multiple-test hypothesis. In the present work, we use FDR [2], since it permits to control the number of False Positives. In a nutshell, the FDR procedure prescribes ordering all p-value from the lowest to greatest, i.e. $\text{p-value}_1 \leq \text{p-value}_2 \leq \cdots \leq \text{p-value}_n$. Then, if n is the total number of tests, the effective threshold is given by the greatest i satisfying

$$\text{p-value}_i \leq i\frac{\alpha}{n},$$

where α is the statistically significant threshold. In the present analysis $\alpha = 0.05$.

6 Validated vs non-validated discursive communities

Let us summarize the procedure for inferring the presence of discursive communities (DiCo) in our dataset, as described in the main text. Our approach focuses on the bipartite network of verified vs. unverified accounts, where a link represents the presence of at least one retweet from the unverified to the verified user. The network is then projected into the layer of verified users, resulting in a monopartite network in which the weights of the link represent the number of common (unverified) retweeters, i.e. the co-occurrences. Finally, the network is validated by comparing the empirical values with a maximum entropy null model (the BiCM [11]), including the information of the bipartite degree sequences.

At first glance, the validation procedure may seem like an unnecessary complication. The goal of the analysis is to extract similarities in the creation of new content based on common audiences, and it can be argued that even without extracting the significant structure of the network, the standard algorithms for community detection can find the relevant network structure.

Before directly comparing the results in the case of our dataset, let us first provide a methodological argument in favor of using the validated projection instead of the entire projection network. As mentioned above, the output of the procedure is a monopartite network in which connections are present if the co-occurrences cannot be explained by the bipartite degree sequences. In this sense, the structure of the network is inferred by discounting the *original* bipartite information. If, instead, the projection network is not validated, the communities in the network are inferred using the information about the projected network, i.e., some kind of information *derived* from the original bipartite system. Note also that knowing the value of the co-occurrences does not allow going back to the bipartite structure of the system and causes a loss of information [6]. In this sense, the use of the original information available from the data should be preferred.

Nevertheless, the implications of such a choice could still be limited in our dataset and, therefore, we will examine the results of the different approaches. The first observation, already highlighted in many papers [1,3–5,7–10], is that, when the debate is political or societal (as in the case of our dataset), the accounts of politicians and political parties tend to cluster, according to their orientation, in the validated network of verified users. This is also the case for our dataset, as can be seen in the left panel of Fig. S5: the colored nodes represent the accounts of political parties and politicians, where the color is related to their political alliance[4]. The only exceptions are some Italia Viva accounts that are merged with some center-left politicians. Such behavior is justified by the fact that Italia Viva was created by politicians who left the PD because they were not satisfied with the current leadership. In this sense, it is not surprising to find links between former party members.

[4]In dark yellow, the Movimento 5 Stelle; in dark blue, the right-wing parties Lega and Fratelli d'Italia; in sky blue, the center-right party Forza Italia; in magenta, the center-left party Italia Viva; in red, the democratic alliance, including PD (the Italian Democratic Party), +Europa, the Socialist Party, and the Green Party. In gray, other verified users whose political orientation is not given *a priori*, such as journalists, media, artists, NGOs, etc.

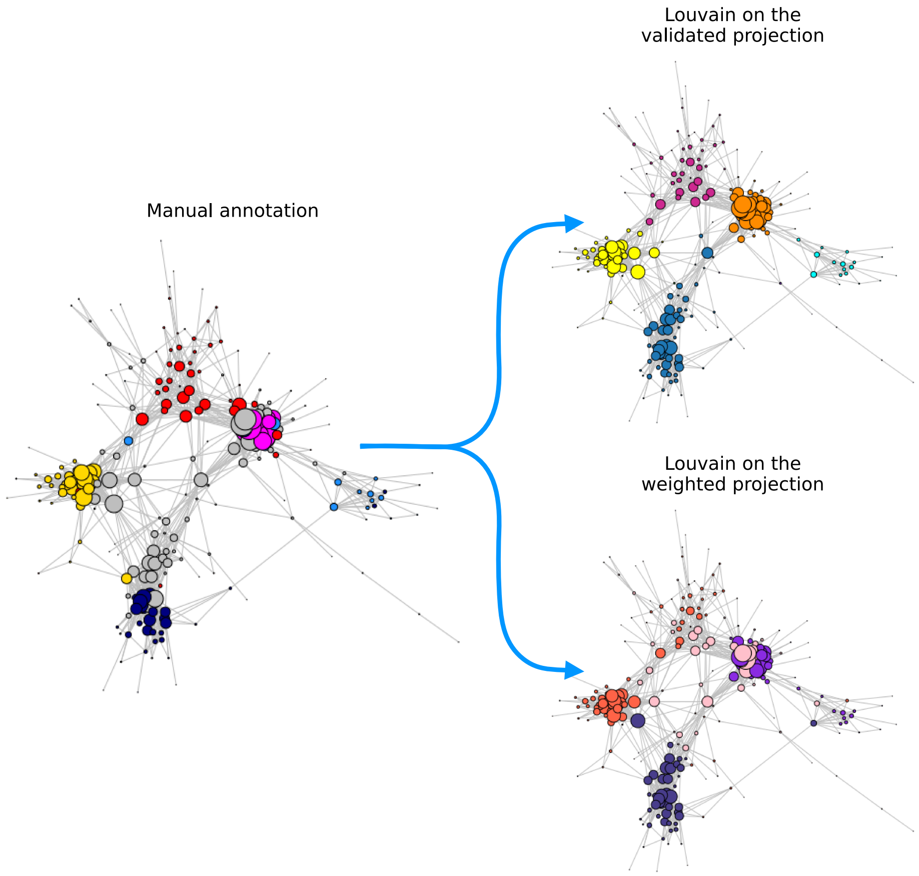

Figure S5: **Comparison between the results of different community detections on the validated network of verified users.** On the left, only politicians' accounts are colored according to their political affiliation (other verified accounts are gray). The first observation is that politicians with similar orientations cluster together in the validated projection. In this sense, a community detection run on this network returns partitions that are coherent with these political clusters (top right panel; nodes with the same color belong to the same community). The same is not quite true for a community detection algorithm run on the non-validated projection: in the latter case, the partitions only partially capture the political orientations present (lower right panel; again, nodes with the same color belong to the same community).

The Louvain algorithm, run on the validated projection, captures such groups, see the top right panel of Fig. S5 (nodes displaying the same colors belong to the same community).

Even if running the (weighted) Louvain algorithm on the entire co-occurrence network yields, by definition, different results, they could still provide a coherent partition of the validated projection, since it represents the core of the co-occurrence network. Remarkably, discounting inferred information has a cost: the obtained partition is less coherent with the political orientations of the verified users than the former one, see the lower right panel of Fig. S5. For example, Movimento 5 Stelle and the center-left alliance are mixed. The situation is even worse for Italia Viva, which is split in 2, partly joining the center-left alliance accounts and partly mixed with Forza Italia. In this sense, we can say that the community detection on the validated projection gives cleaner partitions than

those calculated on the non-validated network. Finally, comparing modularities computed on different types of networks is not particularly informative, but it can still give a rule-of-thumb idea about the organization of the network: in the case of the validated network, the modularity is $Q \simeq 0.66$, while in the case of the non-validated network, it is $Q \simeq 0.17$[5]. In this sense, the validated network has a more modular structure.

In summary, in the validated projection of verified users, politicians and political parties cluster according to their political affiliation, and therefore a community detection algorithm running on the validated projection will capture these groups. Instead, a community detection algorithm running on the entire co-occurrence network of verified users, where co-occurrences is the number of common unverified retweeters, adds some noise to the partition found, and the division between opposing groups is less clean.

References

[1] Carolina Becatti, Guido Caldarelli, Renaud Lambiotte, and Fabio Saracco. Extracting significant signal of news consumption from social networks: the case of twitter in italian political elections. *Palgrave Communications*, 5:1–16, 12 2019.

[2] Yoav Benjamini and Yosef Hochberg. Controlling the false discovery rate: a practical and powerful approach to multiple testing. *Journal of the Royal Statistical Society B*, 57:289–300, 1995.

[3] Matteo Bruno, Renaud Lambiotte, and Fabio Saracco. Brexit and bots: characterizing the behaviour of automated accounts on twitter during the uk election. *EPJ Data Science 2022 11:1*, 11:1–24, 3 2022.

[4] Guido Caldarelli, Rocco De Nicola, Marinella Petrocchi, Manuel Pratelli, and Fabio Saracco. Flow of online misinformation during the peak of the covid-19 pandemic in italy. *EPJ Data Science 2021 10:1*, 10:1–23, 7 2021.

[5] Guido Caldarelli, Rocco De Nicola, Fabio Del Vigna, Marinella Petrocchi, and Fabio Saracco. The role of bot squads in the political propaganda on twitter. *Communications Physics*, 3:1–15, 12 2020.

[6] Jean-Loup Guillaume and Matthieu Latapy. Bipartite structure of all complex networks. *Information Processing Letters*, 90(5):215–221, 2004.

[7] Mattia Mattei, Guido Caldarelli, Tiziano Squartini, and Fabio Saracco. Italian twitter semantic network during the covid-19 epidemic. *EPJ Data Science 2021 10:1*, 10:1–27, 9 2021.

[8] Mattia Mattei, Manuel Pratelli, Guido Caldarelli, Marinella Petrocchi, and Fabio Saracco. Bow-tie structures of twitter discursive communities. *Scientific Reports 2022 12:1*, 12:1–23, 7 2022.

[9] Tommaso Radicioni, Fabio Saracco, Elena Pavan, and Tiziano Squartini. Analysing twitter semantic networks: the case of 2018 italian elections. *Scientific Reports 2021 11:1*, 11:1–22, 6 2021.

[10] Tommaso Radicioni, Tiziano Squartini, Elena Pavan, and Fabio Saracco. Networked partisanship and framing: A socio-semantic network analysis of the italian debate on migration. *PLOS ONE*, 16:e0256705, 3 2021.

[5] Note that the null models implemented by the two Louvain community detection algorithms are different. On the binary validated network, it is the standard binary configuration model, which considers the information of the bipartite degree sequences. On the total co-occurrence network, it is the weighted configuration model, thus including the information of the strength sequence.

[11] Fabio Saracco, Riccardo Di Clemente, Andrea Gabrielli, and Tiziano Squartini. Randomizing bipartite networks: the case of the world trade web. *Scientific Reports*, 5:10595, 9 2015.

[12] Fabio Saracco, Mika J Straka, Riccardo Di Clemente, Andrea Gabrielli, Guido Caldarelli, and Tiziano Squartini. Inferring monopartite projections of bipartite networks: an entropy-based approach. *New Journal of Physics*, 19(5):053022, may 2017.
